# Effect of Potassium Sorbate and Ultrasonic Treatment on the Properties of Fish Scale Collagen/Polyvinyl Alcohol Composite Film

**DOI:** 10.3390/molecules24132363

**Published:** 2019-06-26

**Authors:** Xue Liang, Shiyi Feng, Saeed Ahmed, Wen Qin, Yaowen Liu

**Affiliations:** 1 College of Food Science, Sichuan Agricultural University, Yaan 625014, China; 2 School of Materials Science and Engineering, Southwest Jiaotong University, Chengdu 610031, China; 3 California NanoSystems Institute, University of California, Los Angeles, CA 90095, USA

**Keywords:** potassium sorbate, fish scale collagen, polyvinyl alcohol, ultrasonic treatment, composite films, antibacterial activity

## Abstract

Composite films containing different amounts of potassium sorbate (KS) were prepared by using fish scale collagen (Col) and polyvinyl alcohol (PVA). Fourier transform infrared spectroscopy (FTIR), light transmittance, mechanical, water vapor transmission rate (WVTR), and the antibacterial properties of the composite films were analyzed. The results showed that the addition of Col significantly reduced the light transmittance of the composite film, but KS had no significant effect on the light transmission. The tensile strength decreased first and then increased with the addition of KS, while the WVTR increased first and then decreased. The composite film exhibited a certain degree of antibacterial properties against *E. coli* and *S. aureus*. In addition, we found that ultrasonic treatment reduced the WVTR, and also improved tensile strength and elongation at break of the composite films, but had no significant effect on other properties. The KS/Col/PVA films have the potential to be used as antimicrobial food packaging.

## 1. Introduction

At present, safe and non-toxic degradable packaging materials are research hotspots in the field of food packaging, many researchers use natural biomacromolecules to form different films, such as proteins, polysaccharides, lipids, etc. Collagen can be easily obtained from fish scales, and accounts for 50%-70% of the total mass of fish scales. In addition, the macromolecular spiral structure in fish scales has certain thermal stability and tight fiber structure, which makes the collagen show better toughness, strength and biodegradability. However, the collagen film shows brittleness and solubility in water, which limited its wider application [1]. Polyvinyl alcohol (PVA) is a degradable macromolecular plastic material with good optical properties, high tensile strength and good barrier properties. In addition, PVA has a good application prospect in the field of food packaging [2,3,4]. Due to the defects of fish scale collagen (Col), many papers reported to composite with PVA as the composite film-forming substrate for packaging materials. 

Potassium sorbate (KS) is an unsaturated fatty acid salt of sorbic acid, which can be quickly decomposed by the metabolic system. The concentration of KS has no effect on the flavor of the food and has a good inhibitory effect on mold, yeast, and aerobic bacteria. It is one of the most commonly used antimicrobial agents against fungi, yeast and some bacteria [5,6]. KS has been widely studied in food preservation packaging. Kowalczyk et al. used carboxymethyl cellulose as the substrate emulsion and added KS to the antifungal effect of the composite coated on apricot. It was found that coating with KS is very effective approach against *Botrytis cinerea* and brown rot [7]. It can effectively delay the softening of the apricot and efficiently extend its shelf life. Sousa et al. also found the biodegradable films containing KS could guarantee the microbiological safety of fresh lasagna pastas and avoid the excessive consumption of food additives [8]. Gunaydin et al. added KS as an antifungal agent to the hydroxypropyl methylcellulose-beeswax composite film and studied its effect on the physical and chemical properties of the plums during cold storage. Potassium acid, sodium methylparaben, and sodium hydroxybenzoate composite coated films all showed good preservation effects and did not adversely affect the sensory flavor of the plums and appearance of the fruits [9]. Türe et al. prepared a wheat gluten-resistant antibacterial film containing KS. They found that the composite films showed excellent antimicrobial activity against *Aspergillus niger* [10]. Pranoto et al. fabricated different composite films by adding garlic oil, KS and nisin as antibacterial agents. Adding KS significantly reduced the tensile strength of the composite films, but KS reduced the compactness of the film structure. Because the interaction between the amino group and the carboxyl group of the hydrophilic group in the film inhibits the release of KS, the antibacterial effect of the composite films was weaker than others [11]. Studies have shown that KS can improve the physicochemical, and antibacterial properties of the packaging films [7,8]. Currently, KS is not particularly extensive studied. 

In this study, the fish Col and PVA were used as film-forming substrates, and added KS as an antibacterial agent. Morphology, tensile strength, WVTR, and antibacterial properties of the composite films were discussed in relation to their ingredient concentration. In addition, sonication treatment was used to improve some certain properties of the composite film. 

## 2. Results and Discussion

### 2.1. SEM

**Figure 1:**
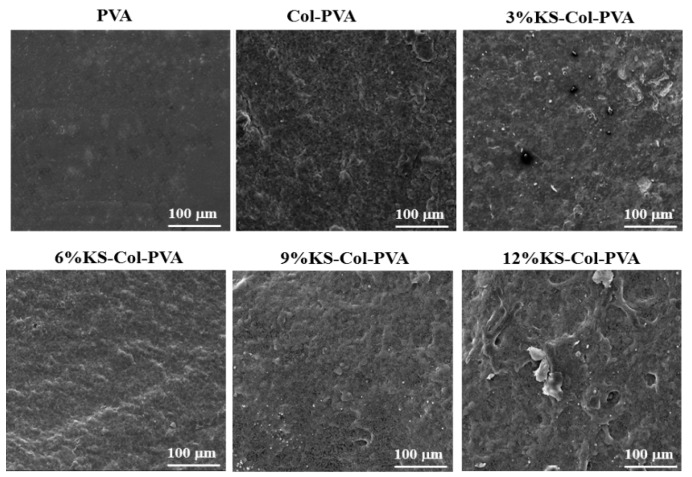
SEM micrographs of the PVA, Col-PVA, and KS-Col-PVA films.

In the present study, the impact of KS addition on material morphology was evaluated by SEM (Figure 1). It can be seen that as the concentration of KS increases, it has a significant effect on morphology of the films. The surface of all samples showed dense, uniform and good film forming properties. The roughness the film gradually increased, and obvious agglomeration occurs especially in 12% KS-Col-PVA film. As the the concentration of KS is too high, it causes uneven dispersion.

### 2.2. Transparency and Color of the Films

**Figure 2:**
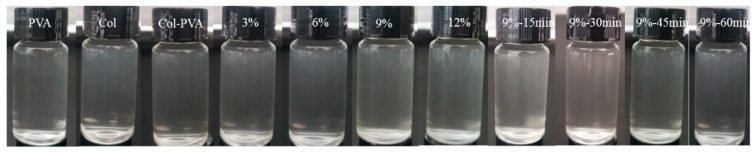
Photograph of composite film solution.

Figure 2 shows the solution with different content of KS. It can be seen that the PVA and Col solution are colorless and transparent. The color of the solution is not obvious when different concentrations of KS were added. 

**Figure 3:**
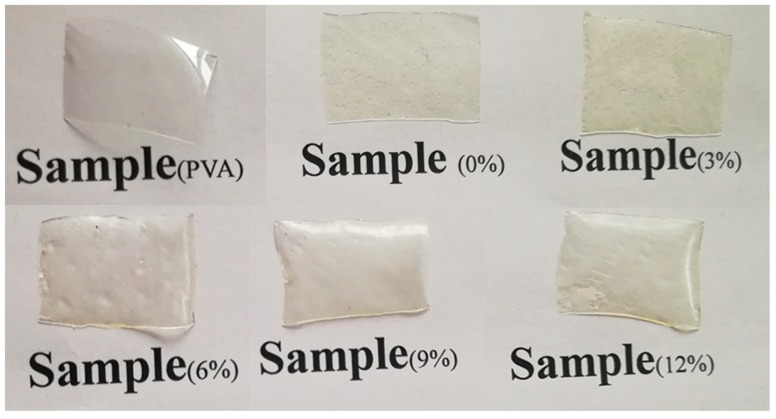
Photograph of PVA and different content KS composite films.

It can be seen from the Figure 3 that the 12% KS-Col-PVA film form more fragments on the surface as SEM image, while other groups have good film-forming properties, and the appearance of the film is uniform and transparent. After adding KS, the surface of the film is rougher than PVA film.

The transparency and color of the packaging material is an important indicator reflecting the appearance of the packaged product. Table 1 shows that the addition of Col caused a significant decrease in the transparency of the composite film (*p* < 0.05), while the addition of KS had a certain effect on the transparency of the composite film, and the difference was not significant (*p* > 0.05). Lopez et al. also proved that the addition of KS has little effect on the light transmission of the composite film [12], so perhaps the addition of collagen reduced the transparency of the composite film. When the content of KS was low, protein and PVA molecules could complete the interaction very well. However, when the content was too high, self-phase gathers and forms crystals will occur to block the passage of light. Table 1 shows the color parameters ΔE of composite films. The addition of KS decreased the L* in the films, leading to a decrease in the transparency of the films. In addition, the presence of KS produced a significant increase in ΔE. The ΔE value of films with Col and KS resulted in a significantly higher value than the control. The ΔE increase was attributed to the increased b*, indicating that active films have more yellow than the control samples due to sorbate oxidative browning [13]. Sousa et al. also mentioned in his research, that this yellowing of the films could be explained by the partial degradation of sorbate by high temperatures employed in the extrusion process, which probably favored the Maillard reaction between the carboxyl group of KS and the matrix of the film [14]. Chevalier et al. also demonstrated that the yellowing could be attributed to the Ti oxidative degradation of KS by cleavage of the double bonds, leading to aldehydes and ketones [15].

### 2.3. Mechanical Properties

The average thickness, tensile strength (TS) and elongation at break (EAB) of the films are presented in Table 1. The TS of pure PVA is (17.4 ± 0.9) MPa, and the addition of Col significantly reduces the TS of the composite film. When the amount of addition reached 9%, the TS of the composite film reached a minimum value of (3.3 ± 0.5) MPa, due to the addition of KS destroying some molecules. The interaction caused the TS of the composite film to decrease, but when the amount of addition was 12%, the TS increased to (3.5 ± 0.8) MPa because of the small molecular substance. When the polar group carboxyl group, hydroxyl group in the KS molecule interact with the amino group and the carboxyl group in the protein molecule to form a physical crosslink, the crosslink between the molecules is tighter, which increases the compactness of the film and reduces the density of the film. Many researchers also reported that different composite films exhibited similar properties in TS after the addition of KS [10,11].

It can be seen in Table 1 that the EAB of the pure PVA film is the best, (14.35 ± 1.11)%. After addition of the Col, the EAB of the composite films decreased significantly (*p* < 0.05), from (14.35 ± 1.11)% to (4.35 ± 0.58)%. With the increase in the amount of KS, an increase in EAB, from (4.35 ± 0.58)% (Col-PVA) to (5.77 ± 0.51)% (9% KS-Col-PVA), occurred. This is due to the interactions between the chains of antimicrobial agents, which penetrate with ease into the film matrix. Consequently, there is greater mobility between the chains, producing films with lower TS and greater flexibility [16,17].

### 2.4. FTIR Analysis

**Figure 4:**
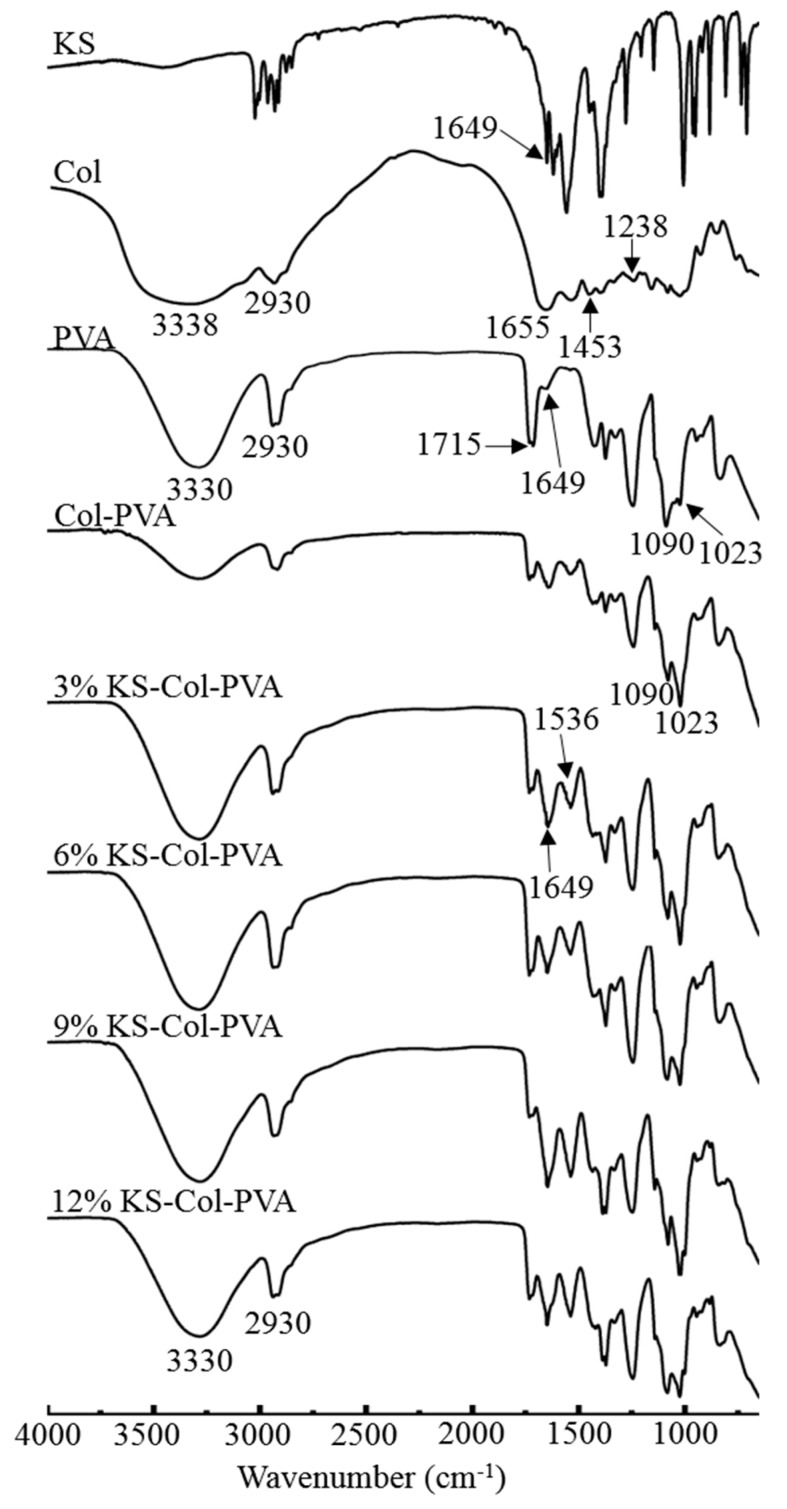
FTIR spectra of the different composite films.

Figure 4 shows that the main functional groups of Col are as follows: 3338 cm^−1^ is the amide A band (-NH stretching vibration), 2930 cm^−1^ is the amide B band (-CH stretching vibration), and 1649 cm^−1^ is the amide I band (C=O stretching vibration and -NH in-plane bending vibration), 1453 cm^−1^ is the amide II band (the absorption peak of the CH_2_- and -COO- bonds), and 1238 cm^−1^ is the amide III band (the stretching vibration of the -NH bond), 1655 cm^−1^ is the stretching vibration of the C=O bond (amide I band) [18]; the characteristic functional group absorption peak of KS is mainly located at the stretching vibration of C=O bond at 1649 cm^−1^. For the characteristic functional group of PVA, the absorption peaks are mainly the stretching vibration absorption peak of the -OH bond near 3300−3400 cm^−1^, the -CH stretching vibration peak near 2900 cm^−1^, and the three peaks of 1750–1500 cm^−1^ from the high wave number to the low wave number, C=O stretching vibration, -OH deformation vibration, and -NH deformation vibration. C-O bending vibration from about 1090 cm^−1^ [19]. It can be seen that after the addition of KS, the peak shape of 3330–3400 cm^−1^, 1536 cm^−1,^ and 1023 cm^−1^ become higher, and had significant changes. Perhaps the increase in the addition of KS caused the increase of -OH radicals in the films. At the same time, it lead to an increase in the absorption peak at the stretching vibration of the C=O bond. In the spectrum of PVA-Col-KS film, a change in the amide I band at 1649 cm^−1^ appears clearly. It is sharper, with the increase of KS incorporated indicating some interaction between a group of PVA [11].

### 2.5. WVTR 

**Figure 5:**
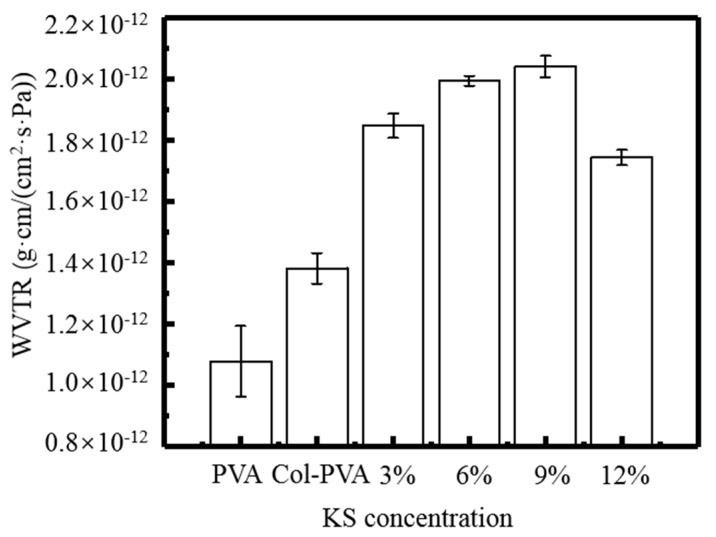
WVTR at different KS content of KS-Col-PVA composite film.

Figure 5 shows that the addition of Col and KS significantly increased the WVTR of the composite films, because the substrate of the film is PVA and Col, and both are rich in hydrophilic groups. In addition, KS is also highly hygroscopic, resulting in a gradual increase in the WVTR of the composite films. Shen et al. also showed a change consistency, and explained that the presence of KS interacts with the molecules of the substrate, changing the network structure in the composite film, thereby affecting the water barrier properties of the composite film [20]. When the amount of KS reached 9%, the WVTR of the composite films reached a maximum of 2.04 × 10^−12^ g·cm/(cm^2^·s·Pa). When added to 12%, the WVTR of the composite films reduced to 1.74 × 10^−12^ g·cm/(cm^2^·s·Pa), perhaps due to the excessive hydrogen bond between the carboxyl group in the KS molecule and the amino group in the protein molecule. The combination increased the compactness of the film, making it difficult for water molecules to diffuse in the film, resulting in a decrease. Many previous studies found that the addition of KS significantly improved the structure of the composite film, affecting the mechanical properties and water barrier properties of the composite films [10,21,22]. 

### 2.6. Antibacterial Testing

In order to investigate the antibacterial effect of KS as an antibacterial agent for the composite films, typical food pathogenic bacteria *E. coli* and *S. aureus* were used as representative bacteria. PVA film has almost no antibacterial properties, and the addition of the Col enhances the antibacterial property of the composite film to a certain extent, but it is still less than 50%. KS enhanced the antibacterial property of the composite film, but the enhancement effect was not obvious. When the KS content is 12%, the antibacterial rate of the composite film against *E. coli* is 69.64%, and the *S. aureus* is 58.59%. Shen et al. also showed that when the content of KS was less than 10%, there was no obvious inhibitory effect on *E. coli*., due to the hydrogen bond interaction between hydroxyl groups of starch and carboxyl groups of KS, which reduced the release of KS around the diaphragm [20]. In addition, it was also found that the inhibitory effect on *S. aureus* was very weak in the whole experimental concentration range of KS. 

**Figure 6:**
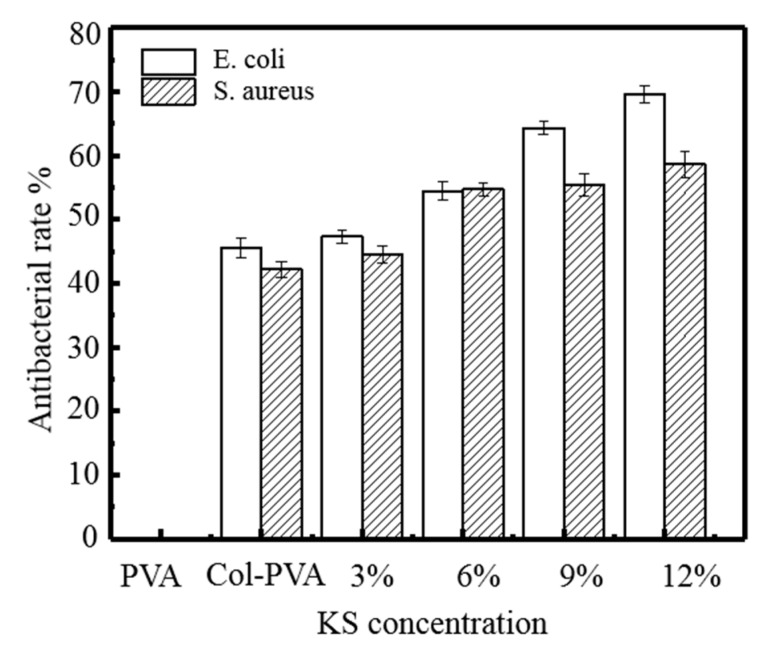
Antibacterial rate of *E. coli* and *S. aureus* at different KS content of KS-Col-PVA composite film.

As can be seen from Figure 6, the antibacterial effect of the composite film on *E. coli* is superior to that of *S. aureus*. As the cell wall lipid content of Gram-negative bacteria is higher, KS molecules are easier to pass, and the cell wall of Gram-positive bacteria contains a large amount of peptidoglycan, which is not easy to pass, thus causing different antibacterial rates against the two bacteria. The antibacterial mechanism of KS could enter the cell through the cell film of the microorganism, causing the pH value in the cell to decrease, changing the film permeability of the cell film or reducing the proton kinetics to destroy the substrate transport, thereby inhibiting the basic metabolic reaction of the cell [20]. In addition, when KS enters the cell, it can bind to the sulfhydryl group in the microbial enzyme system, thereby destroying many enzyme systems and achieving consistent microbial growth and reproduction, thereby exerting antibacterial and antiseptic effects. 

### 2.7. Effect of Ultrasonic Treatment Film Forming Solution on the Performance of the Composite Film

In order to study the effect of ultrasonic treatment of composite films, 9% KS-Col-PVA was selected for ultrasonic treatment at different times (0, 15, 30, 45, 60 min), and the ultrasonic power was 120 W, 50 kHz. After ultrasonic treated, the film was formed on the Teflon plate, and various performance changes were tested.

Table 2 reflects the changes in film properties after ultrasonic treated. It can be seen that with the increase of ultrasonic time, the transparency of the composite films gradually improved. When the ultrasonic time was 30–45 min, the transparency of the composite film reached the minimum value (86.83% ± 0.40%). When the ultrasonic time was 45 min, the TS of the composite film changed significantly, reaching the maximum value of (5.9 ± 0.10) MPa. With the extension of time, the TS and EAB showed a decrease. When the ultrasonic time was 60 min, the TS was (3.4 ± 0.21) MPa and the EAB was (13.02 ± 0.88)%. Ultrasound treatment had no significant effect on the appearance of film, so it was no longer to discuss here. Table 2 also shows that the color of the film changed slightly. With the increase of ultrasonic time, the ΔE value increased first and then decreased. This is due to the more uniform dispersion of KS in the film by ultrasound, which increased the contact between KS and the outside world, thus aggravating sorbate oxidative browning.

**Figure 7:**
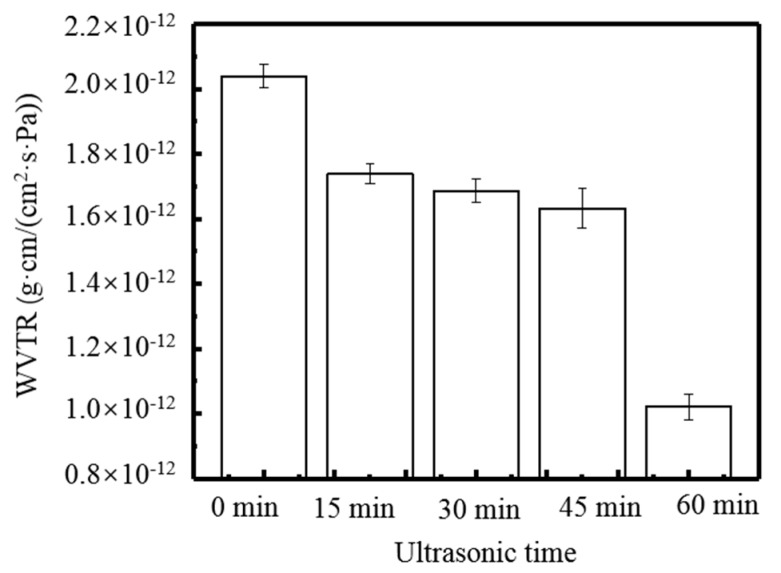
Effect of time of ultrasound treatment of film solution on WVTR.

**Figure 8:**
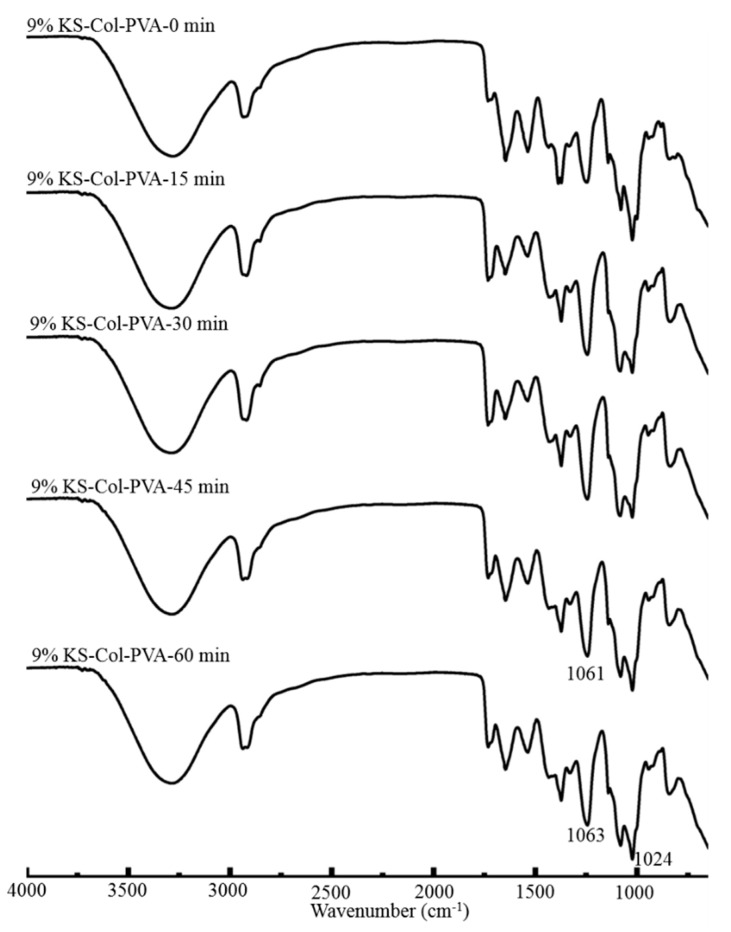
Effect of time of ultrasound treatment of the 9% KS-Col-PVA composite films on FTIR.

**Figure 9:**
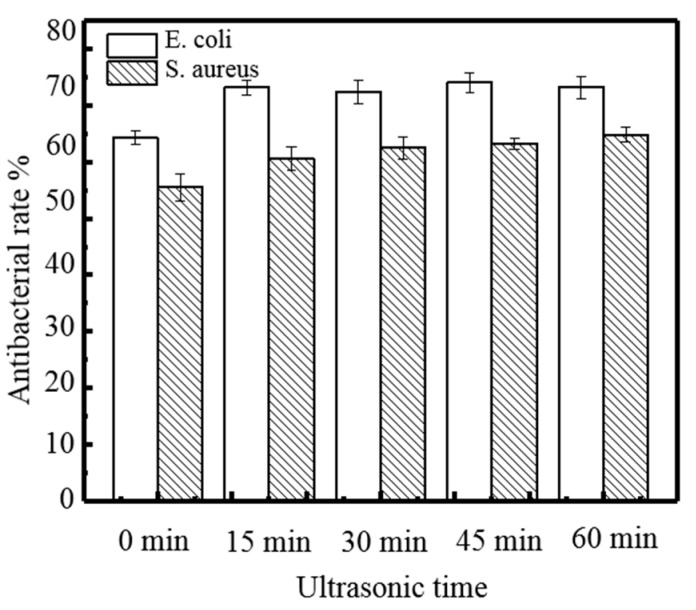
Effect of ultrasound times effect on the antibacterial rate of the film.

Figure 7 shows that the WVTR decreases slightly with ultrasonic time. When time reached 60 min, the lowest WVPR of composite films was 1.02 × 10^−12^ g·cm/(cm^2^·s·Pa). From Figure 8, it can be seen that: 1) the area of the peak at 2930 cm^−1^ increased, 2) the absorption intensity of the peak at 1649 cm^−1^ decreased, and 3) the area of the peak at 1024 cm^−1^ decreased significantly after ultrasonic treatment. The performances of composite films were improved after ultrasonic treatment for a certain time, as the cavitation and super mixing effects of ultrasonic treatment break some chemical bonds in molecules, reduce the size of particles, expose many reaction groups, and facilitate the interaction between molecules. Keeping molecules at a high temperature and pressure makes the interaction between the molecules more rapid and orderly, enhancing the network structure of the film, and thus increasing the transparency of the film [23,24]. Moreover, the ultrasonic treated made the molecules of various substances fully stretched, the TS, EAB, and WVTR of the composite films were significantly improved. When the ultrasonic time increased to 60 min, the transparency of the composite film did not change significantly, but the TS and EAB decreased significantly, and the WVTR increased significantly. This is due to a large number of chemical bonds in the film breaking, making it difficult to form a dense network structure. At the same time, the disordered interaction makes it difficult to form an orderly structure among the molecules, thus weakening the performance of all aspects [21]. Figure 9 shows the antibacterial rate of the composite film increased after ultrasonic treatment, but the effect was small (*p* > 0.05). When the ultrasonic time was 45 min, the maximum antibacterial rate against *E. coli* was 74.11%. When the ultrasonic time was 60 min, the maximum antibacterial rate against *S. aureus* was 64.84%. During the ultrasonic treatment, the molecular chain of each substance in the composite film was gradually broken and exposed, resulting in an increase in the amount of contact with the bacterial cells, thereby causing a slight increase in the antibacterial effect.

## 3. Materials and Methods

### 3.1. Materials

KS and fish Col were obtained from Sigma Aldrich (St Louis, MO, USA). PVA (Mw = 7.6 kDa, Mw/Mn = 1.32) was purchased from Shenzhen Esun Industrial Co., Ltd. (Shenzhen, China). All other chemicals and solvents were grade or higher purity and purchased from Chengdu Kelong Reagent Co. (Chengdu, China) unless otherwise indicated.

### 3.2. Preparation of KS-Col-PVA Composite Films

8 g of Col powder was dissolved in 92 g distilled water, and magnetically stirred at 35 °C water bath for 30 min to obtain a uniform Col solution. Then, 8 g PVA powder was added into 90 g of distilled water and stirred to obtain a bubble-free homogeneous 8% (w/w) PVA solution. 30 g Col solution and 30 g of PVA solution were mixed and magnetically stirred for 60 min [19]. KS powder was weighed and added again to the above Col/PVA mixture solution. Glycerol (2 g/60 g) was used as a plasticizer. Then, 100 g of solution was poured into square plates (20 cm × 20 cm) and dried at 45 °C. Table 3 shows the composition of the different films prepared in this study. All groups have good film-forming properties except the Col solution. Therefore, the Col sample is no longer used and analyzed in subsequent tests.

### 3.3. Scanning Electronic Microscope (SEM)

Micro-structural analysis of composite films was performed using a scanning electron microscope (ZEISS-Supra 55, Jena, Germany). All of composite films were bonded to a conductive carbon tape, coated with Au, and determined by using an accelerating voltage of 20 kV [25].

### 3.4. Transparency and Color of Composite Films

All films were cut into rectangular pieces (10 mm × 30 mm) and placed on the internal side of a spectrophotometer cell. Transparency of films was measured by a spectrophotometer (Model V-530, Jasco International, Tokyo, Japan) at 600 nm. Five replicates of films were tested. The transparency (%) was calculated as the percentual relationship between the light intensity with the specimen in the beam and the light intensity with no specimen in the beam [26]. Color of films were determined with a colorimeter (Minolta chromameter CR-200) using the CIELAB color parameters. L, a, and b values were averaged from nine readings across for each sample on a white background: L = 83.96 ± 0.06; a = 2.05 ± 0.09; b = 1.99 ± 0.07. The total color difference (ΔE) was calculated according to Equation (1):

ΔE = (ΔL*)^2^ + (Δa*)^2^ + (Δb*)^2^ (1)

where ΔL*, Δa* and Δb* are the differentials between a sample color parameter and the color parameters of formulation without Col and without KS used as standard [27].

### 3.5. Mechanical Properties

The mechanical properties of films were obtained at 25 °C The tensile strength (TS) and elongation at break (EAB) of the films were measured by a universal testing machine (UTM, Instron 5583, Instron, Boston, USA). The films were cut into 60 × 10 mm strips, the samples were placed between the tensile grips allowing a grip separation distance of 40 mm with a tensile rate of 10 mm/min. The following properties were calculated from the stress-deformation curves by the following Equations (2) and (3):

TS = F_MAX_/(A × d) (2)

%E = 100 × L/L_0_ (3)

where: F_MAX_ = the pick force at break (N), A = the film thickness (mm), d = the films width (mm), L = the films length at break (mm), L_0_ = the initial film length (mm) [28]. 

### 3.6. Fourier Transform Infrared (FTIR) Analysis 

The interaction among Col, PVA and KS was investigated by a FITR spectroscopy at 25 °C All the samples were recorded on a FT-IR NICOLET 10 spectrophotometer (FT-IR NICOLET 10). The FTIR analysis was performed on the powder state of Col and KS, mixed with KBr powder and palletized. A transmittance mode was used to obtain the FTIR absorption spectrum. The FTIR spectra analysis was performed in the mean infrared region with a wavenumber range of 4000–650 cm^−1^ by averaging 32 scans and spectral resolution of 4 cm^−1^. The experiment was performed twice to verify that the spectra was consistent between individual samples.

### 3.7. Water Vapor Transmission Rate and Water Solubility

The water vapor transmission rate (WVTR) of all the films were evaluated through the procedure described in a previous study of Liang et al. [29]. Briefly, the mouth of a cup containing dry calcium chloride was sealed with the film at 25 °C under 75% relative humidity. The cups were weighed periodically to obtain the mass of moisture transferred through the film into the cup. The WVTR was determined once the weight stopped changing with time and calculated by dividing the slope of the line by the exposed film area on the cup according to Equation (4):

WVTR = Δm/(Δt × A)(4)

where Δm/Δt is the slope (g/s), A is the area of the exposed film surface (m^2^).

The water solubility of KS/Col/PVA films were determined after 24 h of distilled water immersion. Disc-shaped films (approximately 2 cm in diameter) were immersed in distilled water 50 mL) and kept under mechanical stirring by using a shaker at room temperature. After 24 h, samples were dried at 105 °C for 24 h to determine the final dry mass. The solubility, expressed in terms of dissolved dry mass, was determined according to Equation (5):

S = (|m_0_ − m_f_|)/m_0_ × 100% (5)

where S is the water solubility (g/100 g film); m_0_ is the dry mass of the sample (g), and m_f_ is the final mass of the sample after immersion for 24 h (g).

### 3.8. Antibacterial Tests

For the antibacterial tests, *S. aureus* and *E. coli* were selected as Gram-positive and Gram-negative bacteria, respectively. Samples from performed in dynamic shake flask method according to ASTM E2149-10. Bacteria was transferred from stock culture to slant culture medium and then transferred into 5 mL nutritious broth (NB) tube. The tubes were incubated at 37 °C for 24 h and then 1 mL was pipetted into 50 mL NB flasks. The films were cut into pieces and 0.3 g films were added into the flasks and incubated at 37 °C for 24 h. The 10-fold serial dilutions were made by using phosphate-buffered saline 9 mL/tube and 1 mL of each flask pipetted into a dilution tube and diluted continuously to nine tubes. Then, 0.1 mL of bacteria suspension with dilution factor 10^6^–10^8^ was taken into sterilized plates containing 10 mL NB by pipette and plates were incubated at 37 °C for 24 h. All experiments were conducted in triplicate. The colony forming units (CFU) of bacteria were counted, and the antibacterial rate (R) were calculated via Equations (6) and (7), respectively [30]:

CFU/mL = (content of colony × Dilution factor)/(volume of dilution (0.1 mL))(6)

Antibacterial ratio (%) = (A−B) × 100/A.(7)

where A is the CFU of blank sample and B is the CFU of antibacterial samples.

### 3.9. Statistical Analysis

One-way analysis of the variance (ANOVA) was performed using Statgraphics Plus for Windows 5.1 (Manugistics Corp., Rockville, MD, USA). Fisher’s least significant difference (LSD) was used at the 95% confidence level.

## 4. Conclusions

The composite films were prepared by using Col and PVA as the film-forming substrate and KS as the antibacterial agent, and its performance was changed by ultrasonic treatment. The results showed that the addition of Col significantly reduced the light transmittance of the composite film. The ultrasonic treatment improved the transparency and reduced the WVTR of the composite film when ultrasound time reached 45 min. The KS/Col/PVA films have the potential to be used as antimicrobial food packaging.

## Figures and Tables

**Table 1:** Effect of KS concentration on average thickness (AT), moisture content (MC), tensile strength (TS), elongation at break (EAB) and transparency (T) of different composite films.

Sample	AT (mm)	MC (%)	TS (MPa)	EAB (%)	T (%)	Color			
						L*	a*	b*	ΔE
PVA	60.33 ± 2.52^a^	5.9 ± 0.19^a^	17.4 ± 0.9^a^	14.35 ± 1.11^a^	90.87 ± 0.50^a^	85.28 ± 0.16^a^	2.29 ± 0.17^a^	3.74 ± 1.32^a^	1.61 ± 0.16^a^
Col-PVA	57.88 ± 3.79^b^	16.1 ± 2.10^b^	10.9 ± 1.2^b^	4.35 ± 0.58^b^	85.73 ± 2.29^b^	83.38 ± 0.39^b^	2.26 ± 0.15^a^	7.39 ± 2.61^b^	2.50 ± 0.16^b^
3%KS	59.67 ± 5.29^a^	23.3 ± 2.98^c^	6.8 ± 0.5^c^	5.28 ± 0.43^c^	88.70 ± 1.23^a^	84.12 ± 0.16^a^	2.28 ± 0.13^a^	8.67 ± 3.06^b^	2.67 ± 0.09^b^
6%KS	64.33 ± 4.16^c^	17.9 ± 2.22^b^	4.8 ± 0.6^d^	5.43 ± 0.55^c^	87.57 ± 0.95^b^	82.52 ± 1.40^b^	2.32 ± 0.19^a^	9.68 ± 3.42^b^	3.00 ± 0.78^c^
9%KS	60.00 ± 2.31^a^	15.3 ± 1.78^b^	3.3 ± 0.5^d^	5.77 ± 0.51^c^	87.80 ± 1.21^b^	84.08 ± 0.19^a^	2.26 ± 0.15^a^	11.71 ± 4.14^c^	2.78 ± 0.12^b^
12%KS	57.33 ± 3.06^b^	15.2 ± 1.46^b^	3.5 ± 0.8^d^	5.49 ± 0.39^c^	88.93 ± 0.45^a^	84.64 ± 0.10^a^	2.28 + 0.17^a^	13.15 ± 4.65^c^	2.98 ± 0.03^c^

L* a* b* according to the International Commission of Illumination and the YI were determined in at least five different positions for each specimen. Color parameters range from L*=0 (black) to L*=100 (white), -a* (greenness) to +a* (redness) and –b* (blueness) to +b* (yellowness). Data is presented as mean ± standard error and means with a–d different superscript alphabets in the column are significantly different (*p* < 0.05).

**Table 2:** Properties of the film prepared using ultrasound treated 9% KS-Col-PVA composite film (AT: average thickness, MC: moisture content, TS: tensile strength, EAB: elongation at break, T: transparency).

Sample.	AT (mm)	MC (%)	TS (MPa)	EAB (%)	T (%)	Color			
						L*	a*	b*	△E
9%-0	60.00 ± 2.31^a^	15.3 ± 1.78^a^	3.3 ± 0.5^d^	5.77 ± 0.51^c^	87.80 ± 1.21^b^	84.08 ± 0.19^a^	2.26 ± 0.15^a^	11.71 ± 4.14^a^	2.78 ± 0.12^a^
9%-15	57.07 ± 3.61^b^	16.7 ± 1.32^a^	4.5 ± 0.33^b^	14.48 ± 0.92^a^	88.03 ± 0.25^a^	83.89 ± 0.10^a^	2.20 ± 0.10^b^	15.66 ± 5.54^b^	3.12 ± 0.29^b^
9%-30	58.67 ± 1.15^b^	14.2 ± 1.92^a^	5.0 ± 0.52^c^	13.85 ± 0.82^a^	88.00 ± 0.72^a^	82.86 ± 0.77^b^	2.30 ± 0.18^c^	17.53 ± 6.20^c^	3.21 ± 0.28^b^
9%-45	59.33 ± 2.65^a^	13.6 ± 0.98^b^	5.9 ± 0.10^a^	13.13 ± 0.94^a^	86.83 ± 0.40^a^	82.80 ± 1.11^b^	2.24 ± 0.19^a^	18.53 ± 6.55^c^	2.78 ± 0.08^a^
9%-60	57.41 ± 3.46^b^	14.8 ± 0.87^a^	3.4 ± 0.21^a^	13.02 ± 0.88^a^	86.85 ± 0.21^a^	84.25 ± 0.14^a^	2.20 ± 0.10^b^	21.11 ± 6.46^d^	2.93 ± 0.13^a^

L* a* b* according to the International Commission of Illumination and the YI were determined in at least five different positions for each specimen. Color parameters range from L*=0 (black) to L*=100 (white), -a* (greenness) to +a* (redness) and –b* (blueness) to +b* (yellowness). Data is presented as mean ± standard error and means with a–d different superscript alphabets (^a–d^**)** in the column are significantly different (*p* < 0.05).

**Table 3:** Composition of different composite solutions.

Sample	KS (g)	Col (g)	PVA (g)	Gly (g)
Pure-PVA	0	0	4.8	2
Col	0	4.8	0	2
Col-PVA	0	2.4	2.4	2
3%KS-Col-PVA	0.162	2.4	2.4	2
6%KS-Col-PVA	0.324	2.4	2.4	2
9%KS-Col-PVA	0.486	2.4	2.4	2
12%KS-Col-PVA	0.648	2.4	2.4	2
